# Comparison of Two Dimension-Reduction Methods for Network Simulation Models

**DOI:** 10.6028/jres.116.020

**Published:** 2011-10-01

**Authors:** Kevin L. Mills, James J. Filliben

**Affiliations:** National Institute of Standards and Technology, Gaithersburg, MD 20899-0001

**Keywords:** correlation analysis, dimension reduction, network simulation, principal components analysis

## Abstract

Experimenters characterize the behavior of simulation models for data communications networks by measuring multiple responses under selected parameter combinations. The resulting multivariate data may include redundant responses reflecting aspects of a smaller number of underlying behaviors. Reducing the dimension of multivariate responses can reveal the most significant model behaviors, allowing subsequent analyses to focus on one response per behavior. This paper investigates two methods for reducing dimension in multivariate data generated from simulation models. One method combines correlation analysis and clustering. The second method uses principal components analysis. We apply both methods to reduce a 22-dimensional dataset generated by a network simulator. We identify issues that an analyst must decide, and we compare the reductions suggested by the methods. We have used these methods to identify significant behaviors in simulated networks, and we suspect they may be applied to reduce the dimension of empirical data measured from real networks.

## 1. Introduction

Paxson and Floyd [1] describe many difficult problems that impede simulation of large data communication networks, and recommend two main coping strategies: search for invariants and carefully explore the parameter space. Unfortunately, Paxson and Floyd fail to address a key, related question: What model responses should be examined? This question is key because typical network simulation models (e.g., Fall and Varadhan [2], SSFNet [3], Tyan et al. [4], Riley et al. [5], Yaun et al. [6], Zeng et al. [7]) can measure system response through tens to hundreds of outputs, which might represent aspects of fewer significant underlying model behaviors. Usually, experimenters select a subset of model outputs to analyze because considering all available responses proves too time consuming, too costly or computationally infeasible. When choosing a subset of simulation outputs, experimenters using ad hoc selection techniques may omit responses that characterize important model behaviors. Further, experimenters may select outputs in a fashion that overemphasizes particular behaviors. These mistakes become particularly salient during careful exploration of a model’s parameter space, where experimenters seek to understand the response of a model to changes in input parameters.

Overweighting or underweighting significant model behaviors can yield invalid conclusions, thus some method is required to identify precisely the model outputs that correspond to each significant behavior. Fodor [8] describes this mathematically as a dimension reduction problem: “given the *p*-dimensional random variable *x* = (*x*_1_,…, *x_p_*)*^T^*, find a lower dimensional representation, *s* = (*s*_1_, …, *s_k_*)*^T^* with *k* ≤ *p*, that captures the content in the original data, according to some criterion.” Fodor goes on to survey numerous linear and non-linear techniques that may be applied to reduce the dimension of high-dimensional data sets. Adopting any of these techniques would provide a principled approach that experimenters could use to identify significant model behaviors from a large collection of model output data. Of course, one wonders whether some techniques are superior to others. Fodor identifies principal components analysis (PCA) as the best (in terms of mean-square error) linear dimension reduction technique

In this paper, we combine correlation analysis and clustering (CAC) to identify significant behaviors in MesoNet [9], a network simulation model implemented in SLX^1^ [10]. We also use PCA as a basis to identify an alternative view of significant behaviors. In applying each method, we identify issues that an analyst must decide and we compare the behaviors identified by the methods. The paper makes three contributions: (1) describes and applies two methods to identify significant behaviors in network simulators, (2) identifies specific judgments that must be made by an analyst when applying each method and (3) compares the dimension reductions achieved by the two methods. The ideas contained in this paper facilitate effective reduction in response dimension for large simulations and should improve the ability of researchers and practitioners to analyze results from simulation experiments.

The paper is organized in six main sections. In Sec. 2 we explain the general idea underlying dimension reduction for multivariate responses, as applied to simulation outputs. In Sec. 3 we identify and summarize candidate MesoNet responses, showing how those responses form a multivariate dataset. MesoNet is described more fully elsewhere [9]. Section 4 applies correlation analysis and clustering (CAC) to identify significant MesoNet behaviors, while also identifying decisions an analyst must make. Section 5 applies principal components analysis (PCA), along with key analyst decisions, to produce two alternate views of significant MesoNet responses. In Sec. 6 we compare and contrast the significant behaviors found by CAC and PCA, we discuss pros and cons of the two methods, and we give our reasons for preferring the reduction proposed by CAC. We conclude in Sec. 7.

## 2. Dimension Reduction for Multivariate Responses

As illustrated in Eq. (1), a simulation model can be viewed as a function transforming a set of *n* input parameters, *x*_1_ to *x_n_*, into a set of *m* responses, *y*_1_ to *y_m_*. Each input parameter can take on a range of values, e.g., 1 to *k*, defining a parameter space of size *k^n^*, which can be very large. Elsewhere [11] we explain methods to provide significant information while simulating only a reduced number (2*^n−r^*) of parameter combinations. Here, we focus on methods to reduce the number of responses that must be analyzed, while still reflecting the most significant model behaviors.

(1) $$y_{1},\ldots ,y_{m}=f(x_{1|[1,\ldots ,k]},\ldots ,x_{n|[1,\ldots ,k]}).$$

In this paper, we apply two methods that can reduce dimensionality of a model response space: correlation analysis combined with clustering (CAC) and principal components analysis (PCA). First, CAC suggests a reduction in the number of responses from *m* to *z*_1_. We use domain expertise to select *z*_1_ specific responses from among the responses shown in Table 1. Second, PCA suggests a reduction from *m* to *z*_2_. We use heuristics to select *z*_2_ specific responses from Table 1. Subsequently, we compare the specific responses selected by each method.

## 3. Overview of Candidate MesoNet Responses

For purposes of our case study, we selected 22 candidate responses from among those generated by MesoNet, a data communications network simulator. The first sixteen responses (y1 – y16), in Table 1 characterize various aspects of network-wide behavior. The flow of data traffic in the network is regulated with the transmission control protocol (TCP), which requires a source and receiver to exchange connection packets, after which the source sends a flow of data packets to a receiver, who responds with acknowledgments. Inability to exchange connection packets prior to a deadline causes a connection attempt to fail. For successful connections, the rate of transmission on a flow is controlled by a congestion window (*cwnd*) that defines the number of packets a source may transmit before receiving an acknowledgment. The time between sending a data packet and receiving an acknowledgment is known as the round-trip time, which is composed of propagation delays on network links plus queuing delays incurred when waiting in routers for transmission. In general, TCP flows begin with a low *cwnd* and then increase the *cwnd* as acknowledgments arrive. Failure to receive acknowledgments leads to a timeout, which causes a source to retransmit unacknowledged data packets. Similarly, receipt of a negative acknowledgment, indicating a specific packet loss, will also stimulate retransmission by a source. Responses y5 – y16 measure most aspects of these processes involved in transmitting data between sources and receivers. Responses y1 – y4 measure macroscopic load on the network: the number (and proportion) of sources transmitting data packets and the rate at which data packets enter and exit the network.

**Fig. 1:**
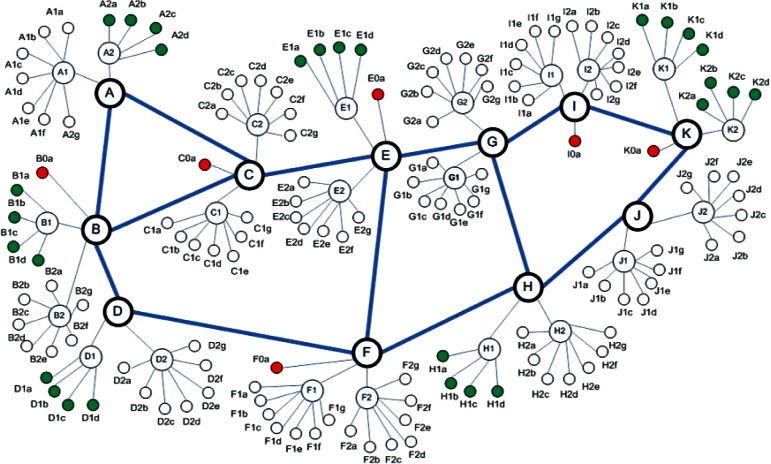
Example Three-Tier MesoNet Topology.

The remaining six responses (y17 – y22) measure the average throughput achieved on flows transiting specific types of paths in a network topology, such as illustrated in Fig. 1. MesoNet topologies consist of three router tiers–backbone, point of presence (PoP) and access–providing transit paths for sources and receivers connected in a fourth tier (not visible in Fig. 1). The speed of various router classes can be computed relative to a network speed, *s*, which is one of the input parameters of MesoNet. Backbone routers operate at a speed of 2*s* and POP routers operate at 0.25*s*. Access routers, which constrain transmission capacity available to sources and receivers, can be one of three types: (1) **D**-class, connecting directly to the network backbone and operating as fast as POP routers, (2) **F**-class, operating at a speed of 0.05*s* and sharing a POP router with three other access routers, and (3) **N**-class, sharing a POP router with six other access routers and operating at the slowest speed (0.025*s*) among all access routers. Flows enter and exit the topology at access routers, thus access routers may be used to classify flows into **DD**, **DF**, **DN**, **FF**, **FN** and **NN** flows, where the flow class denotes the potential throughput (e.g., **DD** flows can achieve higher throughput than **DF** flows and so on).

**Fig. 2:**
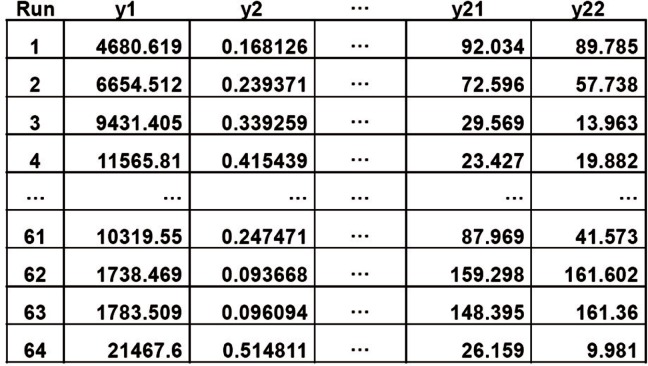
Sample (Partial) Multivariate Dataset Reflecting 22 Responses for each of 64 MesoNet Simulations.

We generated a 64 × 22 multivariate dataset (Fig. 2) by running 64 simulations, where each simulation consisted of different combinations of values for 11 of the 20 MesoNet parameters. Values for the remaining nine parameters were fixed in all runs. For each simulation we measured the 22 responses given in Table 1. We used a 2^11–5^ orthogonal fractional factorial design [12] to ensure that we simulated a balanced arrangement of parameter combinations. Next we apply two different methods to reduce the dimension of the data in Fig. 2.

## 4. Dimension Reduction via Correlation Analysis and Clustering

**Fig. 3:**
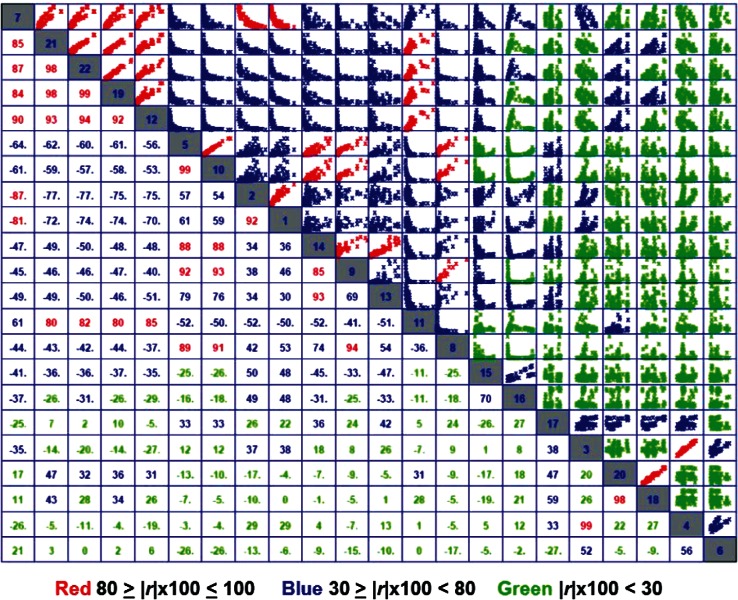
Pair-wise Correlation Matrix: scatter plots above diagonal, correlation coefficient (|*r*| × 100) below diagonal.

**Fig. 4:**
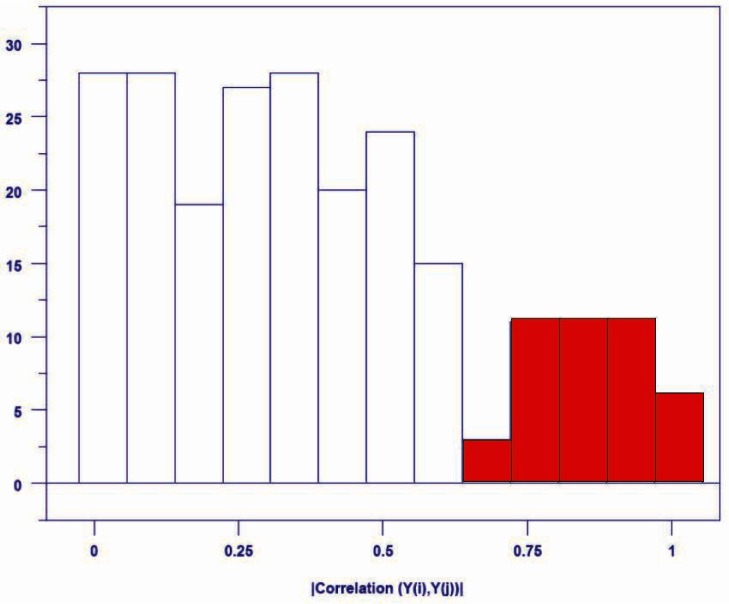
Frequency Distribution of |*r*| for pair-wise correlations; |*r*| > 0.65 highlighted in red.

As a preliminary step, we computed the correlation coefficients (*r*) for each pair of the 22 responses, and summarized the results in a matrix (Fig. 3), which gives 231 pair-wise scatter plots in cells above and to the right of the diagonal and corresponding correlation coefficients (scaled × 100) below and to the left of the diagonal. We color the scaled correlations: above 80 red, below 30 green and intermediate values blue. We order the matrix by decreasing value of mean correlation for each response. In Fig. 3, response y7 (gray cell labeled 7 in the upper left hand corner) has largest mean correlation and response y6 (gray cell labeled 6 in lower right hand corner) has smallest mean correlation. Cells on the diagonal identify the response related to cells in the corresponding row and column. For example, consider the gray cell labeled 13 (response y13) in the middle of Fig. 3. The column of cells moving upward gives scatter plots for y13 and each of the 11 responses (y9 – y7) higher on the diagonal; the row moving leftward gives the corresponding correlation coefficients. The row of cells moving rightward from 13 gives scatter plots for y13 and each of the 10 responses (y11– y6) lower on the diagonal; the column moving downward gives the corresponding correlation coefficients. Given pair-wise correlations, an analyst must decide which pairs to include in further analysis and which pairs to discard. To help with this decision, we plot a frequency distribution (Fig. 4) of the absolute values, |*r*|, of correlation coefficients for all response pairs. We use the frequency plot to select a threshold for correlations to consider further. Here, we chose |*r*| > 0.65 because the histogram shows a notable change in pattern above that value, appearing as a separate (sub)distribution of 42 pair-wise correlations centered on a different mode.

**Fig. 5:**
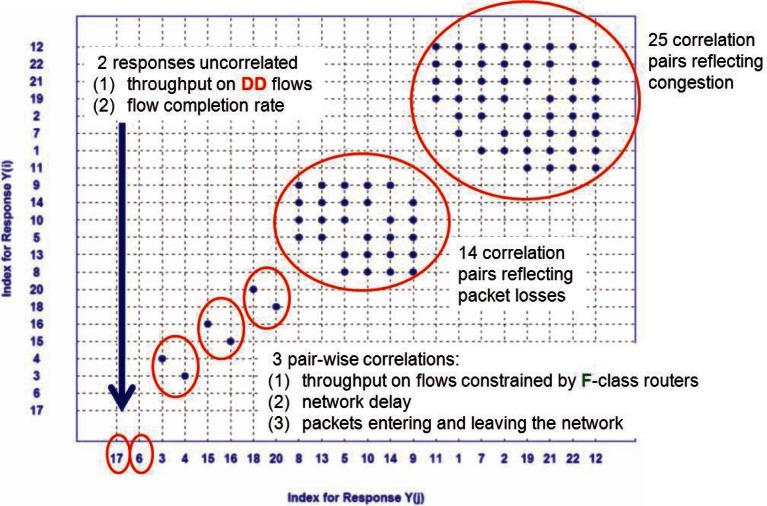
Index-Index plot sorted by increasing count of correlation pairs to identify clusters of mutual correlations.

Next, we construct an index-index plot, where both the x and y axes indicate the index of corresponding responses (1–22). We plot a blue dot for each of the 42 *y_i,j_* pairs where |*r*| > 0.65. We identify the response that is correlated with the most other responses and create a self-contained subset from those responses. We then repeat the process for those responses remaining outside the subset, forming a second self-contained subset. We continue repeating the process until all responses have been allocated to a subset. Subsequently, we reorder the axes of the index plot so that response identifiers are arrayed in increasing order of the cardinality of the subset of which they are a member. Response identifiers within each subset are ordered arbitrarily. Figure 5, which shows the resulting sorted index-index plot generated from the 42 correlation pairs selected from Fig. 3, reveals five subsets (or clusters) of mutually correlated responses, and two responses that were not correlated with any others. The largest cluster (25 correlation pairs) includes responses that reflect network congestion. The second largest group (14 correlation pairs) includes responses reflecting packet losses. Three pair-wise correlations reflect: (1) throughput on flows constrained by **F**-class access routers, (2) network delay and (3) packets entering and leaving the network. One uncorrelated response (y17) measures average throughput on **DD** flows, while the other (y6) measures flows completed per second. We summarize these response groupings under the CAC half of Table 2, which identifies seven significant behaviors, a reduction of 15 dimensions. For each behavior that includes multiple responses, an analyst must select one response to represent the behavior.

We used domain-specific reasoning to select the responses highlighted in bold under the CAC half of Table 2. Regarding congestion, response y22 measured throughput on the most numerous flows in the simulation, which transited the most congested paths, so all aspects of network congestion were likely to influence this response. Regarding losses, response y10 measured the retransmission rate on all data packets, which would be caused by losses of data, acknowledgments and negative acknowledgments, and so would be approximately twice the loss rate (y5) on data packets. Negative acknowledgements (y13) would be stimulated by lost data packets but could also be lost, as could acknowledgements. Timeouts (y14) would occur only for severely disrupted packet flows. Connection failures (y8 and y9) would measure only the influence of lost connection-establishment packets. For these reasons, we decided that response y10 should give the most comprehensive reflection of packet losses. Regarding delay, the smoothed round-trip time (y15) would be influenced by both propagation and queuing delay, while response y16 reflects only queuing delay, so we selected y15 as a more comprehensive measure of delay. Regarding **F**-class throughput, we selected response y20 (throughput on **FF** flows) because the related flows do not transit any **N**-class or **D**-class access routers, which should allow **FF** flows to give the best representation of **F**-class throughput. Regarding packet throughput, we reasoned that packets could not leave the network (y4) unless they were injected (y3), so we decided that y4 could represent both the rate of packets injected and influence of other factors on the rate of packets leaving the network. Responses y17 and y6 each represent an additional dimension.

The PCA half of Table 2 indicates the responses grouped by PCA into the first four principal components, as explained below in Sec. 5. As we will show, selecting a response to represent each PC relies on a choice among heuristics that have no specific domain interpretation. For this reason, we will compare PC response selections made by two different heuristics. In Sec. 6, we compare the responses we selected from the CAC half of Table 2 against responses representing PCs, as identified by the two heuristics we used. We used one of those heuristics to identify the responses highlight in bold in the PCA half of Table 2.

## 5. Dimension Reduction Based on Principal Components Analysis

Principal Components Analysis (PCA) [8,13–14] is a statistical technique which transforms a set of possibly correlated variables (such as the 22 variables identified in Table 1 and analyzed above in Sec. 4) into an equal number of orthogonal (and hence uncorrelated) variables called principal components (PCs). Each principle component is a weighted linear combination (LC) of the original variables. The first principle component (PC1) is that weighted LC with maximum spread (variance) when the data is projected onto that LC. The second principle component (PC2) is that LC which has the largest projected-data variance of all LCs that are orthogonal to the first PC. The third principle component is that LC which has the largest projected-data variance of all LCs that are orthogonal to the first 2 PCs, and so forth. The last principle component (PC22) is that LC which has the largest projected-data variance of all LCs that are orthogonal to all prior PCs. The 22 PCs thus provide an alternative orthogonal axis system that spans the entire 22-dimensional data space in an ordered fashion–from most explanatory variation down to least explanatory variation.

There are several variations of PCA-depending on (1) the nature of the input data (i.e., raw or untransformed data versus transformed data), and on (2) the nature of the computationally intermediate variation matrix (i.e., covariance matrix versus correlation matrix). The PCA analysis selected in our study used the raw data (normalized to mean 0 and standard deviation 1, in order to enhance comparisons) and the correlation matrix of such data. The computational result of the PCA process is a square matrix–22 × 22 in our case–where the first column contains the weight vector for PC1, the second column contains the weight vector for PC2 and so on. The process also yields a vector containing the progressively-decreasing standard deviations accounted for by each PC. Table 3 shows (a) the standard deviation vector and (b) part of the matrix of weight vectors computed for our case study.

Given the matrix of weights and the vector of standard deviations, an analyst must choose how many PCs to use. An analyst might also decide which variables should be retained in each PC, because PC expressions may be simplified by ignoring the (low weight) contributions of certain of the variables. If the retained set of variables for the chosen PCs includes all or most of the variables, then an analysis might seek to further reduce the set of variables to include only a handful of the most important. To choose the number of PCs to use, one credible source [14] suggests including the first *n* PCs that account for between 70 % and 90 % of the total variance in the data. Examining the cumulative % column of Table 3(a) suggests that the first four PCs should be used (as we did in Table 2) and that PC5 might be used. In Sec. 6, when comparing the responses we selected using CAC against the responses recommended by PCA, we will consider PC1 through PC7, because the CAC suggested seven dimensions were required to represent system behavior, even though the PCA inclusion heuristic suggests that only the first four or five PCs capture sufficient variation in the response data.

**Fig. 6:**
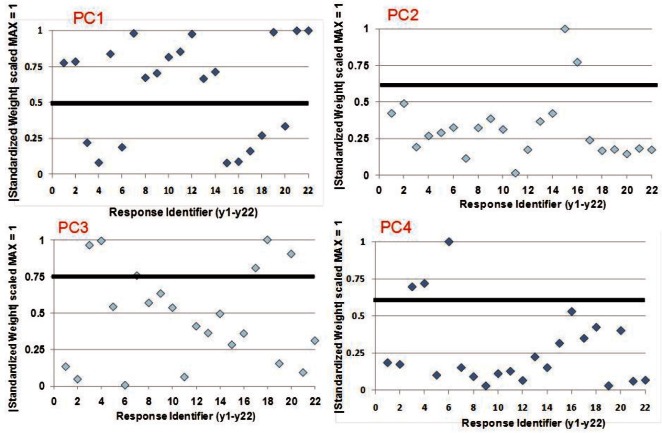
Plots of normalized and scaled weight vectors (y axis) against response variable identifiers (x axis) for the first four PCs. Each plot includes the visually selected threshold (black horizontal line) used to identify responses to include (above the line) and exclude (on or below the line) for each PC. For each plot, responses with normalized and scaled weights above the selected threshold were included for the corresponding PC in the PCA half of Table 2.

We tried several different statistical heuristics to prune variables from PCs. While some provided clearer results than others, all the heuristics generally pointed toward the same outcome. We obtained the most clear cut results using the following heuristic: (1) normalize the weights to zero mean and unit standard deviation, (2) take the absolute value of the normalized weights, (3) scale the results to the range 0 to 1, (4) plot the results, (5) visually identify a separating threshold and (6) use responses plotted above the threshold. Figure 6 shows the application of this heuristic to the first four PCs, yielding the four groupings shown in the PCA half of Table 2. What remains is to select specific responses to represent the PCs of interest.

While one could choose to base subsequent data analyses on the first four PCs, interpretation of such analyses can be difficult because PCs do not necessarily represent any distinguishable domain concepts (though we attempt to supply a mapping to domain concepts in Table 2). As an alternative to using the first four PCs, we can instead select a subset of the variables. Jolliffe [14] suggests that the number of variables to select should equal the number of PCs used, or perhaps a few more. Since the PCA analysis identified that we should use four or five PCs, we decided to select seven variables, which matches the number of variables we selected using CAC.

Selecting specific variables requires an additional heuristic. We tried two particular approaches in order to compare the outcomes. One heuristic, which we denote as MKB, described by Mardia, Kent and Bibby [15], iterates over the weight vectors from the least significant (PC22 here) to the most significant (PC1). When examining each weight vector, the response corresponding to the weight with the largest magnitude is discarded from further consideration. This process continues until the remaining number of responses corresponds to the number (seven here) of variables sought. This seemingly counterintuitive algorithm appears to be based on the assumption that responses that contribute large weights to insignificant PCs must not be particularly salient with respect to the dataset.

The second heuristic we used, which we denote here as inverted MKB, was derived by inverting the MKB algorithm. The inverted MKB algorithm iterates over the weight vectors from the most significant to the least significant. When examining each weight vector, the variable corresponding to the weight with the largest magnitude is withdrawn from further consideration. This process continues until the cardinality of the set of withdrawn responses equals the number of variables sought. The set of withdrawn responses constitutes the responses to be used in subsequent analyses. Table 4 shows the results of applying these two heuristics. The table also shows the seven responses we selected based on CAC. We compare and contrast these results in the next section.

## 6. Comparing Dimension Reduction Methods

In Sec. 5, applying a heuristic threshold to the cumulative distribution of the proportion of variance led us to conclude that the first four PCs accounted for most of the significant variance in our dataset. As shown in Table 2, we were able to develop a domain interpretation of PC1 through PC4,^2^ and we could relate that interpretation to the seven dimensions identified using CAC. The results show relatively good alignment: PCA merged congestion and losses into a single dimension and merged throughput on flows constrained by either **D**-class or **F**-class routers. On the other hand, PCA spread packet throughput among two PCs, rather than retaining it as a unique dimension. In related work [16], guided by analysis of the responses grouped into PC1-PC4, we were able to analyze and interpret the PCs themselves in one situation. In a second situation, we were unable to construct a domain interpretation of even the top four PCs. In situations where PCs cannot be interpreted successfully, one can resort to substituting a set of responses for the PCs. Here, guided by Table 4, we compare the responses identified by the MKB and inverted MKB heuristics against each other and against the responses selected based on a domain analysis of the CAC results. We consider up to seven responses because CAC identified the need for seven dimensions to cover the response dataset.

The top four responses selected by the inverted MKB heuristic can be interpreted to represent congestion (y22), delay (y15), **D**- and **F**-class throughput (y18) and flows completed per second (y6). Under such an interpretation, the inverted MKB heuristic maps nicely to the first four PCs of Table 2. Indeed, three of the first four responses selected by the inverted MKB heuristic matched responses selected by domain analysis of the CAC results. Three of the top four responses selected by the MKB heuristic can be interpreted to represent congestion (y19), **D**- and **F**-class throughput (y18) and flows completed per second (y6). Interpreting the remaining response (y4) is more problematic because y4 appears in two PCs in Table 2, and both PCs have already been covered by other responses. Considering only the top four responses, the inverted MKB heuristic gave results that were better aligned with the CAC results.

Expanding our comparison with the CAC results, we consider additional responses (five through seven) produced by each heuristic. The MKB heuristic identifies connection failure rate (y9), which can be interpreted as representing losses, **DD**-flow throughput (y17), which can be interpreted as representing **D**-class throughput, and y16, which can be interpreted as representing delay. In addition, as its first response, the MKB heuristic identifies y4, which corresponds to packet throughput. Under this interpretation, the top seven responses identified by the MKB heuristic correspond with the seven dimensions identified by CAC. The inverted MKB heuristic also identifies y17 (**D**-class throughput) and y16 (delay) in its top seven responses. However, the inverted MKB heuristic had already included a response (y15) representing delay, so y16 appears redundant. In addition, the inverted MKB heuristic includes response y11 (congestion window size), which can be interpreted to provide a duplicate response representing congestion. Given the two duplicates, the inverted MKB heuristic does not include responses representing losses or packet throughput. Of course, the PCA grouped losses together with congestion and spread packets output across two PCs, so the results of the inverted MKB heuristic are consistent with the PCA response groupings, but not consistent with the seven CAC dimensions. The MKB heuristic appears more consistent with the seven CAC dimensions. On the other hand, considering only the top four responses, the inverted MKB heuristic provides a better correspondence with the CAC analysis.

The PCA-based method provided greater dimension reduction (22 → 4) than CAC (22 → 7). On the other hand, because PCs are uncorrelated variables created from a set of possibly correlated variables, PCA guarantees no obvious domain interpretation of even the top 2 or 3 PCs,^3^ though in the case discussed in this paper we were able to arrive at a reasonable interpretation. In other cases (not described here) we were unable to infer any convincing domain interpretation. Even when a reasonable domain interpretation is possible, PCs may take on both positive and negative values for which domain analysts cannot determine any obvious interpretation, even after establishing a meaning for a PC itself. For example, based on the specific responses that showed significant weights for PC1, we inferred that the PC represented congestion (including its influence on packet losses). Determining whether positive or negative values for the PC represented higher or lower congestion proved quite challenging [16]. To make such a determination we had to compare the parameter settings causing positive and negative PC values with the parameter settings for individual responses that were heavily weighted in the PC. Since we could easily interpret the domain meaning of the concrete values produced for the responses, we were usually able to infer seemingly plausible mappings to positive and negative PC values. While in some situations such chains of inference may be possible, we remain uneasy about the validity of the resulting interpretations. To overcome these limitations with respect to PCs, we adopted two different heuristics to identify responses to substitute for the most significant four PCs. One heuristic (inverted MKB) identified four responses that were within the set of seven responses identified using CAC, and identified a fifth response that could be mapped to a response identified using CAC The other heuristic (MKB) identified four responses that were within the set of seven responses identified using CAC, and identified seven responses that could be mapped to responses identified using CAC. We have no rigorous criteria for judging which of the two heuristics produced results better aligned with the CAC results. Further, the general success of such heuristics is not guaranteed. In fact, we suspect that such heuristics might prove less successful when no convincing domain interpretation can be established for the PCs.

The CAC method provided effective dimension reduction through correlations that could be vetted easily by domain experts. Further, examining pair-wise response correlations helped to validate that MesoNet provides reasonable behavior as a simulation model for data communications networks. The CAC method allowed us to uncover the nuanced difference between network-wide throughput in terms of packets per second versus flows completed per second, a difference that might otherwise have been overlooked. In addition, the CAC method allowed us to discern significant differences in factors influencing the throughput of **DD** flows, as compared to other **D**- and **F**-class flows. These differences were masked by the coarseness of the PCs identified using PCA. On the other hand, when CAC was applied to analyze data from a second simulation experiment (see Appendix C in Ref. [16]) using the same parameters set to different values, the method identified some differences in response correlation, when compared with those shown in this case study. While these differences could be justified by domain analysis, the existence of such differences indicates that separate correlation analyses must be generated and examined for each set of simulation experiments conducted. We found this also true for PCA.

In the end, the CAC method proved more useful to domain analysts seeking to understand output from the MesoNet simulation. The CAC method identified and clustered correlated variables, whereas the PCA method constructed artificial uncorrelated variables that did not necessarily have a domain interpretation. The clusters identified by CAC uncovered some nuanced differences in network behavior, differences concealed within the coarser PCs produced by PCA. Further, the clustered correlated variables identified by CAC could be used in validating that MesoNet produced behavior reasonably aligned with a real network. The PCs produced by PCA could not be used for model validation. In addition, in applying CAC, an analyst need only make one heuristic decision: the threshold for correlation values to include in the clustering step. Domain analysis is used to select which response to adopt as a surrogate for each cluster containing multiple responses. In applying PCA, an analyst needs heuristics to decide: (1) which PCs to include, (2) which responses should be included within each PC, (3) whether PCs should be analyzed directly or whether surrogate responses should be selected, and (4) if surrogate responses are needed, how to identify which responses to use. The necessity to make four heuristic decisions (vs. one heuristic decision for CAC) leads to greater burden for an analyst, provides increased room for error and necessarily affects the repeatability of the PCA method.

## 7. Conclusions

We investigated CAC and PCA as two techniques to reduce the dimension of multivariate response data produced from simulation models. We applied each method to reduce a 22-dimensional response space generated by MesoNet, a simulator for data communications networks. While the PCA method usually suggests a smaller number of dimensions, we found that the CAC method distinguishes dimensions that better correspond with a domain analyst’s understanding of the domain. Further, the results from CAC are much easier for a domain analyst to interpret, which aids in model validation. On the other hand, PCA and CAC appear to be reasonably complementary methods that, applied together, can provide alternate views of a multivariate data set. Comparing dimensions identified by both methods (as we did, for example, in Table 2) should provide a better understanding of responses from simulation experiments. Where only one method is used, we suggest that CAC provides response groupings that are straightforward for a domain analyst to interpret. Using only PCA leads to response groupings that are coarse, abstract and often difficult for a domain analyst to understand. No matter which method is applied, each new data set generated by different experiments with a given simulation model must be subjected to a separate analysis and interpretation because correlations and PCs may change with changes in parameter values.

## Figures and Tables

**Table 1:** Candidate MesoNet Responses

Response	Definition
y1	Active Sources–sources attempting to transfer data
y2	Proportion of total sources that are active: y1/All Sources
y3	Data packets entering the network/second
y4	Data packets leaving the network/second
y5	Packet Loss Rate: y4/(y3 + y4)
y6	Flows Completed per second
y7	Flow Completion Rate: y6/(y6 + y1)
y8	Connection Failures per second
y9	Connection Failure Rate: y8/(y8 + y1)
y10	Retransmission Rate (ratio)
y11	Average Per Flow Congestion Window (packets)
y12	Average Number of Increases in Congestion Window/flow/second
y13	Average Number of Negative Acknowledgments/flow/second
y14	Average Number of Timeouts/flow/second
y15	Average Round-trip Time (ms)
y16	Relative Queuing Delay (y15/average propagation delay)
y17	Average Throughput (packets/second) for **DD** Flows
y18	Average Throughput (packets/second) for **DF** Flows
y19	Average Throughput (packets/second) for **DN** Flows
y20	Average Throughput (packets/second) for **FF** Flows
y21	Average Throughput (packets/second) for **FN** Flows
y22	Average Throughput (packets/second) for **NN** Flows

**Table 2:** Response Groupings by Correlation Analysis and Clustering (CAC) and by Principal Components Analysis (PCA)–as explained in the text, responses highlighted in bold (seven for CAC and four for PCA) were chosen to represent each grouping

CAC Dimension	Responses	PCA Dimension (PC)	Responses
Congestion	y1, y2, y7, y11, y12, y19, y21, **y22**	Congestion	y1, y2, y5, y7, y8, y9, y10, y11, y12, y13, y14, y19, y21, **y22**
Losses	y5, y8, y9, **y10**, y13, y14		
Delay	**y15**, y16	Delay	**y15**, y16
**F**-class Throughput	y18, **y20**		
**D**-class Throughput	**y17**	**D**-class & **F**-class Throughput	y3, y4, y17, **y18**, y20
Packet Throughput	y3, **y4**		
Flows Per Second	**y6**	Flows Per Second	y3, y4, **y6**

**Table 3:** (a) Standard deviation vector resulting from PCA in our case study, along with a cumulative distribution of % variance and (b) elided matrix of vectors (one per PC), where each vector gives the weight (rounded) assigned to each of the 22 responses

PC	(a) Distribution of Variance				(b) Weight Vector Matrix				
	Std. Dev.	Cumulative %	Response	PC1	PC2	PC3	PC4	…	PC22
PC1	9.7091	0.441325	y1	0.26	0.14	−0.17	0.11	…	−0.02
PC2	4.0161	0.62388	y2	0.26	0.18	−0.14	0.11	…	−0.00
PC3	3.2322	0.77079	y3	0.09	0.02	−0.41	−0.39	…	0.70
PC4	2.0630	0.86457	y4	0.04	0.06	−0.43	−0.40	…	−0.69
PC5	0.9716	0.90873	y5	0.28	−0.23	0.04	0.06	…	−0.06
PC6	0.7585	0.94321	y6	−0.04	0.09	−0.12	−0.56	…	0.01
PC7	0.4537	0.96383	y7	−0.28	−0.14	0.10	−0.08	…	−0.01
PC8	0.2569	0.97551	y8	0.23	−0.25	0.04	0.06	…	−0.05
PC9	0.1835	0.98385	y9	0.24	−0.28	0.06	0.02	…	0.01
PC10	0.1254	0.98955	y10	0.27	−0.24	0.03	0.07	…	0.04
PC11	0.0588	0.99222	y11	−0.25	−0.08	−0.11	0.08	…	0.00
PC12	0.0504	0.99451	y12	−0.28	−0.17	−0.00	0.04	…	−0.01
PC13	0.0360	0.99615	y13	0.22	−0.27	−0.02	−0.12	…	−0.00
PC14	0.0286	0.99745	y14	0.24	−0.30	0.02	−0.08	…	−0.02
PC15	0.0225	0.99847	y15	0.04	0.44	−0.04	0.19	…	0.01
PC16	0.0113	0.99899	y16	0.05	0.32	−0.23	0.31	…	0.02
PC17	0.0099	0.99944	y17	0.07	−0.20	−0.37	0.21	…	−0.03
PC18	0.0060	0.99971	y18	−0.06	−0.16	−0.43	0.25	…	0.05
PC19	0.0032	0.99985	y19	−0.29	−0.17	−0.08	0.02	…	0.04
PC20	0.0019	0.99994	y20	−0.08	−0.15	−0.40	0.24	…	−0.06
PC21	0.0011	0.99999	y21	−0.29	−0.17	−0.10	0.04	…	0.07
PC22	0.0002	1.00000	y22	0.26	−0.17	−0.03	0.03	…	−0.07

**Table 4:** Columns two and three give the ordering of response significance identified from PCA weight vectors using two heuristics. The first four responses correspond to the number suggested by analysis of the cumulative distribution of the proportion of PC variance. The fifth response corresponds to a fifth PC, which was on the borderline for inclusion. The sixth and seventh responses correspond to two additional dimensions (PC6 and PC7). The fourth column repeats, for comparison, the specific responses identified by a domain analysis to represent each of the seven clusters of correlated responses using CAC

PC	Inverted MKB Heuristic	MKB Heuristic	CAC Domain Analysis
PC1	Y22 (**NN** flow TP)	Y4 (packets output)	Y22 (**NN** flow TP)
PC2	Y15 (SRTT)	Y19 (**DN** flow TP)	Y15 (SRTT)
PC3	Y18 (**DF** flow TP)	Y18 (**DF** flow TP)	Y4 (packets output)
PC4	Y6 (flows completed TP)	Y6 (flows completed TP)	Y6 (flows completed TP)
PC5	Y11 (CWND)	Y9 (connection failure rate)	Y10 (retransmission rate)
PC6	Y17 (**DD** flow TP)	Y17 (***DD*** flow TP)	Y20 (**FF** flow TP)
PC7	Y16 (queuing delay)	Y16 (queuing delay)	Y17 (**DD** flow TP)
PC8	Y13 (NAKs)	Y13 (NAKs)	
PC9	Y1 (active sources)	Y11 (CWND)	
PC10	Y14 (timeouts)	Y15 (SRTT)	
PC11	Y2 (% sources active)	Y7 (flow-completion rate)	
PC12	Y9 (connection failure rate)	Y2 (% sources active)	
PC13	Y10 (retransmission rate)	Y10 (retransmission rate)	
PC14	Y8 (connection failures)	Y8 (connection failures)	
PC15	Y7 (flow-completion rate)	Y14 (timeouts)	
PC16	Y12 (CWND increases)	Y12 (CWND increases)	
PC17	Y21 (**FN** flow TP)	Y1 (active sources)	
PC18	Y20 (**FF** flow TP)	Y21 (**FN** flow TP)	
PC19	Y19 (**DN** flow TP)	Y20 (**FF** flow TP)	
PC20	Y4 (packets output)	Y22 (**NN** flow TP)	
PC21	Y5 (loss rate)	Y5 (loss rate)	
PC22	Y3 (packets input)	Y3 (packets input)	

## Footnotes

1 Certain commercial equipment, instruments, or software are identified in this paper to foster understanding. Such identification does not imply recommendation or endorsement by the National Institute of Standards and Technology, nor does it imply that the software or equipment identified are necessarily the best available for the purpose.

2 We were unable to devise a domain interpretation for PC5 – PC7, which provides additional evidence that only the first four PCs should be used in this case study.

3 Note, however, that PC1 will almost always represent some kind of “global average”.
